# Solution Based Methods for the Fabrication of Carbon Nanotube Modified Atomic Force Microscopy Probes

**DOI:** 10.3390/nano7110346

**Published:** 2017-10-25

**Authors:** Ashley D. Slattery, Cameron J. Shearer, Joseph G. Shapter, Jamie S. Quinton, Christopher T. Gibson

**Affiliations:** 1 Flinders Centre for NanoScale Science and Technology, College of Science and Engineering, Flinders University, Bedford Park, SA 5042, Australia; Ashley.slattery@adelaide.edu.au (A.D.S.); joe.shapter@flinders.edu.au (J.G.S.); jamie.quinton@flinders.edu.au (J.S.Q.); 2 Adelaide Microscopy, The University of Adelaide, Adelaide, SA 5005, Australia

**Keywords:** atomic force microscopy, carbon nanotubes, dielectrophoresis

## Abstract

High aspect ratio carbon nanotubes are ideal candidates to improve the resolution and lifetime of atomic force microscopy (AFM) probes. Here, we present simple methods for the preparation of carbon nanotube modified AFM probes utilising solvent evaporation or dielectrophoresis. Scanning electron microscopy (SEM) of the modified probes shows that the carbon nanotubes attach to the probe apex as fibres and display a high aspect ratio. Many of the probes made in this manner were initially found to exhibit anomalous feedback characteristics during scanning, which rendered them unsuitable for imaging. However, we further developed and demonstrated a simple method to stabilise the carbon nanotube fibres by scanning with high force in tapping mode, which either shortens or straightens the carbon fibre, resulting in stable and high quality imaging AFM imaging.

## 1. Introduction

Carbon nanotubes (CNTs) have been recognised as the ultimate probe for use in many atomic force microscope (AFM) applications, due to their small diameter, high aspect ratio and wear resistance. These features of CNT probes mean that they can substantially improve the quality of data produced by AFMs such as spatial resolution, force resolution (in force spectroscopy) and for metrology applications where the high aspect ratio nature of CNT probes is particularly advantageous [1,2]. Recent work has also shown that carbon nanotube probes can be very effective for accurate determination of thickness of two-dimensional 2D nanomaterials such as graphene [3], and in high-resolution conductivity imaging of surfaces [4].

There are limited sources of commercially available CNT modified probes and these are typically expensive (>USD 100, e.g., FN-1 series K-Tek Nanotechnology). Therefore, researchers are still seeking cheap and reliable methods to prepare CNT modified probes in-house. Strategies for CNT attachment have included direct CNT growth on the silicon AFM tip by chemical vapour deposition (CVD) [5,6], manual attachment using optical or electron microscopy [7,8], pick-up from CNT-covered surfaces [9,10,11], and dielectrophoresis [12,13,14,15], among others [16]. Reviews by Wilson et al. [1] and Slattery et al. [2] provide excellent overviews of the many fabrication methods and applications of CNT probes.

Apart for the attachment procedure itself, what has been particularly challenging is that even after a CNT has been successfully attached to an AFM probe there is no guarantee that it will be able to effectively perform various AFM measurements. This is because the CNT, while attached to the AFM tip, may still be too unstable to perform AFM measurements and some further methods must be followed to further stabilise the CNT so that it can acquire usable data. Chang-Soon et al. [17] reported that multi-walled CNT (MWCNT) fibres attached to AFM probes straightened and aligned in the direction of a focused ion beam (FIB), and used this to improve the imaging stability of their fibres. FIB irradiation was also used to align CNT fibres as reported by Shin et al. [18] and Raghuveer et al. [19]. Both groups observed that TEM images of the CNT after FIB irradiation showed significant damage to the graphitic structure and substantial implantation of metal ions. In this work we present simple methods for the preparation of carbon nanotube modified AFM probes utilising solvent-based deposition which can be divided into dielectrophoresis and solvent evaporation approaches.

Scanning electron microscopy (SEM) of the CNT probes using these methods shows that the carbon nanotubes attach to the probe apex as fibres and display high aspect ratio. Probes made using these solvent based methods were, in many cases, initially found to exhibit anomalous feedback characteristics during AFM scanning. Therefore we also developed and demonstrate a simple method to stabilise the carbon nanotube fibres by scanning surfaces with high force in tapping mode which either shortens and/or straightens the carbon fibre which results in stable imaging.

## 2. Results

### 2.1. Production of CNT Fibre Probes Using Dielectrophoresis

When a polarisable particle immersed in a medium is subjected to a non-uniform electric field, the particle becomes polarised and experiences a force. This is known as a dielectrophoretic (DEP) force and it causes the particle to align either with or against the field lines, depending on the particles’ permittivity. The DEP force is dependent on a number of factors including dielectric properties of the particle and medium, the size and shape of the particle and the frequency of the applied electric field. DEP has found extensive applications in nanotechnology, as it offers the ability to selectively align and assemble nano and micro-particles based on their size, shape and electrical properties. DEP assembly has also been used by several groups to attach CNTs onto sharpened tungsten tips, as the application of a heterogeneous alternating current between two electrodes immersed in a CNT solution results in the growth of a long CNT fibre [20,21].

Excellent reviews by Pethig and Zhang et al. [22,23] cover the theory of DEP and a range of applications to which this effect has been applied.

**Figure 1:**
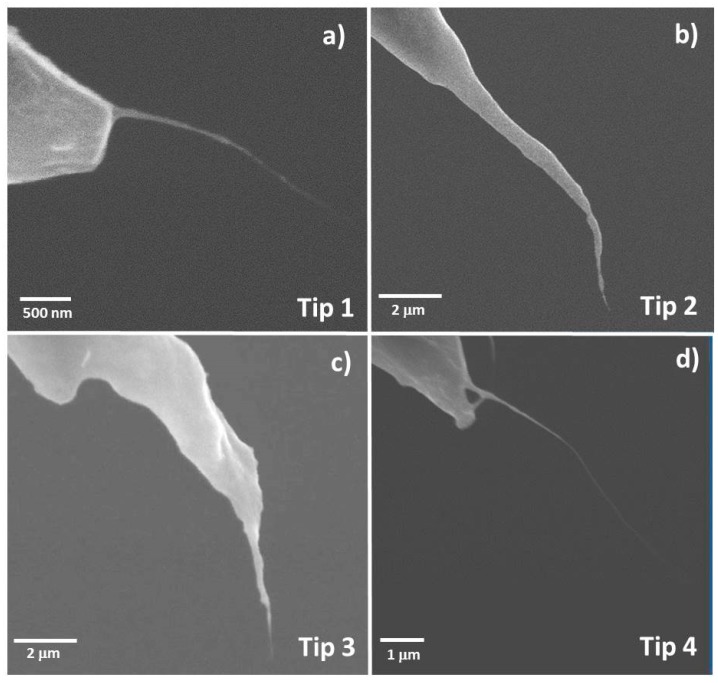
(**a**–**d**) 4 unique CNT tips fabricated by the DEP technique with 2.5 V potential, 1 MHz modulation and 20 s deposition.

Figure 1 shows examples of AFM probes that have been modified with CNT fibres using DEP. We achieved a success rate of greater than 90% in modifying the probe with a long CNT fibre. Unfortunately, the alignment of the fibre was often far from ideal, with many bending away from the tip orientation or exhibiting significant curvature near the end. This is likely due to a number of factors, including the alignment of the tip to the silicon surface, force from the meniscus as the tip is removed from solution, and internal stress on the fibre as the solvent dries. Occasionally, longer CNT fibres were observable by the optical microscope and these were observed to slightly after removal from the solution, presumably due to solvent evaporation from the fibre. Most of these factors would be difficult to avoid, although improvement of the tip alignment would be possible with a more powerful optical microscope and greater control over tilt and positioning. Nevertheless, the prepared CNT modified probes show significantly improved aspect ratio as compared to the AFM tips that they are adhered to.

### 2.2. Production of CNT Fibre Probes Using Solvent Evaporation

**Figure 2:**
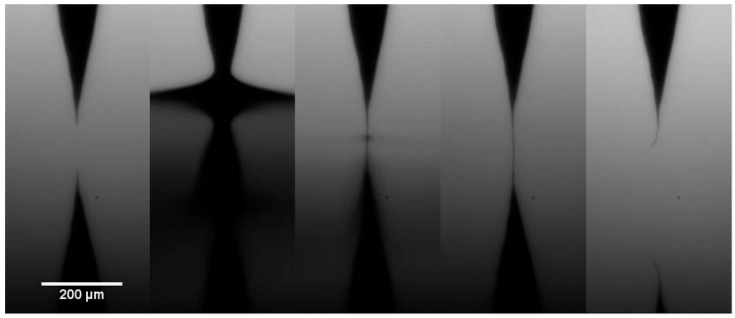
Sequential optical images (left to right) of the tungsten tip and silicon surface, immersed in CNT solution, showing evaporation of the CNT solution and formation of a nanotube fibre with no applied bias.

The other approach used is a simple evaporation technique, which is yet to be reported for CNTs. Fibre formation was observed to occur during the DEP process, but without any applied bias. This intriguing result was further investigated to ensure that there was indeed no potential difference across the gap, by electrically grounding both the tip and the surface. Growth of the fibre was observed using an optical microscope, as shown in Figure 2. An etched tungsten wire was initially used as an ideal, sharp tip which was positioned some distance from the surface and a droplet of the CNT solution was applied to completely immerse the tip. Evaporation of the volatile tetrahydrofuran (THF) solution occurred rapidly, resulting in the meniscus withdrawing over the tip and leaving a thin fibre attached.

A similar process was reported by Ondarcuhu and Joachim, where a gold nanoparticle solution was used to form nanoparticle fibres [24]. This appears to be the only report of fibre formation at a sharp tip in the absence of any electrical bias, and is thus the first report of this technique being applied to CNT solutions. There has been development in CNT fibre production by wet-spinning CNTs from superacid solutions (i.e., sulfuric acid) or utilising polymer stabilisers through a narrow aperture for high throughput production [25,26,27,28,29].

The results presented here differ in that the solution used is not a hazardous superacid, and the fibre is formed at a sharp tip instead of through a nozzle. The closest report to this type of fibre formation is that by Zhang et al. [29] who forced an etheylene glycol dispersed solution of MWCNTs through a syringe nozzle, into a solution of ether. This technique was used to form fibres 10–80 µm in diameter, which were free from any surfactants. The main difference to the technique presented here is the use of single-walled CNTs (SWCNTs) and that the formation mechanism (using evaporation at a sharp tip) is entirely different.

There has been much experimental and theoretical work on the alignment of nanomaterials (including CNTs) at receding solvent interfaces [30,31]. These prior studies provide a good understanding of the mechanisms underpinning formation of the fibre, which is often referred to as the “coffee stain” effect [32]. Preferential evaporation at the solid-liquid-air interface causes the CNTs to be transported via liquid flow to the edge of the solvent and the increase in concentration results in the CNTs ordering with nematic liquid crystal structure. As the droplet recedes over the tip apex, it is proposed that the CNTs order inside the capillary neck which then evaporates and leaves a highly-ordered fibre.

**Figure 3:**
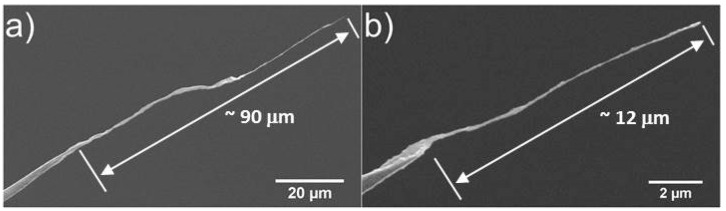
Scanning electronic microscopy (SEM) images showing the variation in fibre length with increased tip-sample separation from (**a**) 100 µm to (**b**) 400 µm.

**Figure 4:**
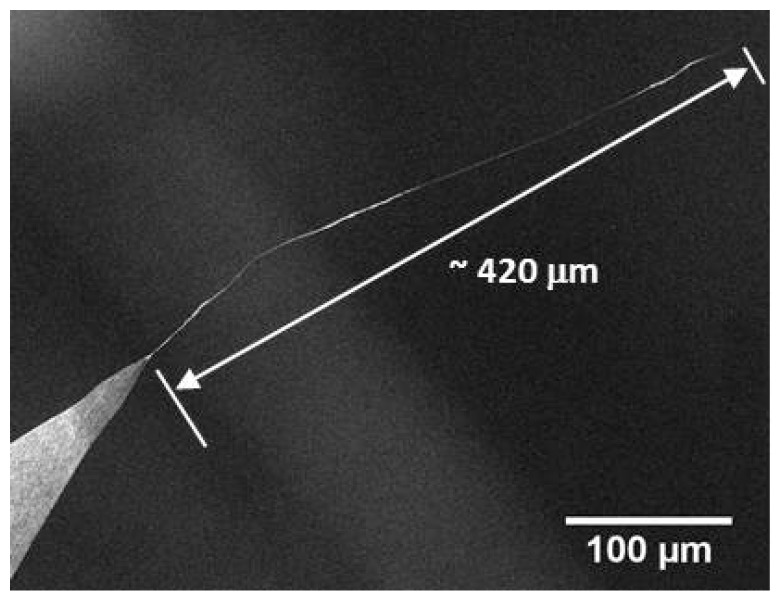
SEM image of a long CNT fibre, drawn by the solvent evaporation method.

Several factors were observed to affect the properties of the nanotube fibre including the tip-sample separation, tip geometry and concentration of the CNT solution. This effect is attributed to the correlation between tip-sample separation and the withdraw rate of the tip from the solution. The receding rate of the droplet was observed to decelerate as evaporation proceeded, resulting in the withdrawal speed of the tip being greater for increased tip-surface distances. One would also expect the concentration of the solution to increase as the droplet evaporated, which makes this factor difficult to control. The length of the fibre was observed to be inversely proportional to the tip-surface separation, resulting in shorter fibres forming for greater separations. This is shown in Figure 3 where a separation of 100 µm yielded at 90 µm fibre and a separation of 400 µm yielded a 12 µm fibre. It was also possible to draw fibres which that were extremely long; Figure 4 shows a CNT fibre that was almost half a millimeter in length (~420 µm).

It is expected that with optimal drawing conditions and a large reservoir of fluid, the fibre could be drawn continuously. Macroscopic CNT fibres have many applications such as flexible conductors for textiles and batteries, as mechanical actuators for muscles [33] and field emission sources for vacuum electronic instruments such as SEMs [21]. This solvent based method could be used in such applications.

**Figure 5:**
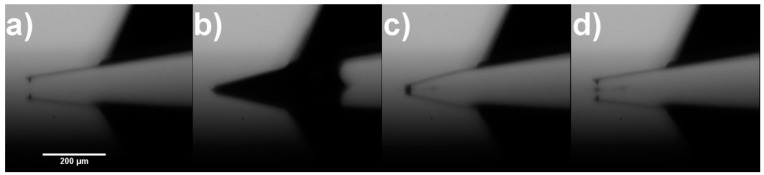
Optical images showing alignment of an atomic force microscope (AFM) tip with a silicon surface (**a**) and the behavior of the cantilever due to evaporation of the CNT solution (**b**–**d**).

The same process can be applied to an AFM tip; however, the cantilever introduces some non-uniform geometry in comparison to the symmetrical etched tungsten tips. The cantilever is placed a certain distance from the silicon surface as shown in Figure 5a, and the droplet is then applied over the entire tip; Figure 5b–d shows gradual evaporation of the solvent and deposition of the fibre. The cantilever was generally flexible enough that the capillary force pulled the tip into contact with the surface, which would be expected to interfere with fibre formation. It can be seen, however, that the cantilever returns to its original position after the droplet has evaporated. Once the droplet has receded sufficiently, to the point that the meniscus force is lesser than the restoring force of the AFM cantilever, the tip will be pulled away from the surface and fibre formation should occur as expected.

**Figure 6:**
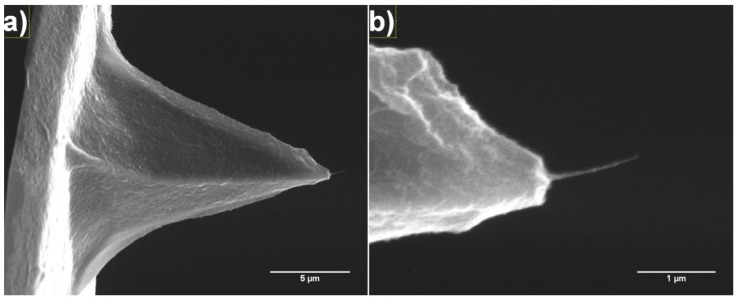
SEM images of a CNT fibre attached to the apex of an AFM probe from the solvent evaporation method showing (**a**) low and (**b**) high magnification of the same tip.

An SWCNT fibre deposited on the AFM probe is shown in Figure 6 and is approximately 1 µm in length. The slightly curved geometry is commonly observed for fibres formed using solution-based methods as observed previously for DEP deposition. Similar to the DEP method we achieved a success rate of CNT fibre deposition greater than 90%.

### 2.3. Fibre Processing and Stabilisation

For the probes displayed in Figure 1, the CNT fibres were, initially, too mechanically unstable to obtain images and required shortening and/or alignment with the axis of the AFM tip. As previously described, this can be achieved using methods such as ion beam irradiation. However, it was found that imaging with high tapping force (or low amplitude set-point) in tapping mode could also stabilise the CNT fibre which is achieved using an AFM instrument.

A detailed description of this stabilisation procedure is given in Section 4.4 but is briefly described here. The AFM probe was engaged in tapping mode and the set-point reduced to 10–30% of its free amplitude; this was continued until the probe started to track the surface properly (this could be 1–20 min), at which time the amplitude set-point was increased to a normal operating value which would be typically 70% to 80% of the cantilever free amplitude. Some additional images may be acquired to allow the CNT fibre to “settle” into a completely stable configuration. This amplitude set-point reduction approach is similar to a method used by Gibson et al. for attaching CNTs with the pick-up method [9]. 

**Figure 7:**
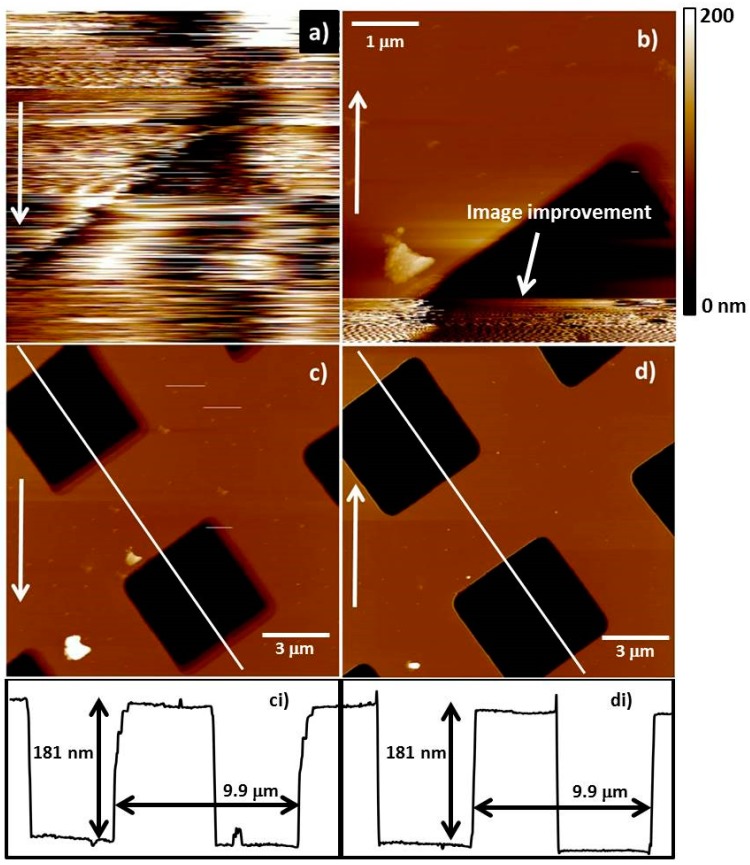
The stabilisation process can be seen in this figure where a CNT fibre probe is scanning a silicon calibration grid (Bruker model number VGRP: 10 µm pitch, 180 nm depth). The image is scanned with 10–30% of free amplitude (**a**) until a clearer image is obtained (**b**) and then the free amplitude is increased to ~70%. With further imaging the CNT continues to align and imaging improves (**c**,**d**). The white arrow in each of the images denotes the direction the tip was scanning the surface in the slow scan axis.

An example of this stabilisation process can be seen in Figure 7, where a CNT fibre probe is scanning a silicon calibration grid (Bruker model number VGRP: 10 µm pitch, 180 nm depth). The white arrow in each of the images denotes the direction the tip was scanning the surface in the slow scan axis. i.e., for Figure 7a the arrow indicates the tip is moving from the top to the bottom in the slow scan axis. Figure 7a is a 5 × 5 micron image and demonstrates the lack of probe stability as the noise level is exceptionally high (hundreds of nanometers) and an accurate image of the surface is impossible to discern. For Figure 7b the bottom portion of the image (~15%) is undergoing the stabilisation procedure and an image of the surface can now be observed indicating some improvement in the alignment of the CNT fibre. Figure 7c,d are larger images (15 × 15 microns) with Figure 7c showing an image with vastly improved quality compared to Figure 7a with the cross section in Figure 7ci showing the correct height and pitch for this model of calibration grid. Figure 7c however does display some “double” tip artifacts on the edge of the grids. This is improved further in Figure 7d, which does not show tip artifacts and, as can be seen if Figure 7di, also shows the correct dimensions for the silicon calibration grid.

**Figure 8:**
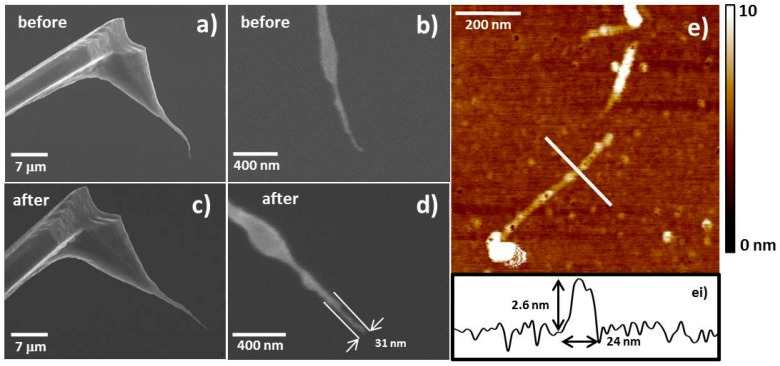
SEM images of tip 2 before processing (**a**,**b**) and after processing (**c**,**d**). Figure 8e is an AFM image of a CNT on a CNT covered silicon surface using tip 2 after processing with Figure 8ei showing a cross section which corresponds to the white line in Figure 8e. Comparing the before and after SEM images indicates there has been a significant amount of straightening of the CNT fibre.

Detailed SEM analysis was performed on tips 1 to 4 in Figure 1 before and after CNT fibre processing to observe what can happen to the CNT fibre after stabilisation has occured. The results for successful stabilisation can be seen in Figure 8 (tip 2 in Figure 1) and Figures S1 and S2 (tips 1 and 3 in Figure 1) in the supplementary material. Each of these figures shows SEM images of the CNT fibre probes before processing (Figure 8a,b, Figures S1a,b and S2a,b) and after processing (Figure 8c,d, Figures S1c,d and S2c,d). AFM images of a CNT covered silicon surface (grown on the silicon surface using CVD and described in Section 4.4) are also shown in Figure 8e, Figures S1e and S2e and were acquired to demonstrate the stable imaging of the CNT fibre probes.

As can been in Figure 8, Figures S1 and S2, the stabilisation process can result in a significant straightening (Figure 8) of the CNT fibre, a subtle straightening (Figure S1) or a shortening of the CNT fibre (Figure S2). All resulted in high aspect ratio CNT fibre probes capable of acquiring high quality AFM images. The approximate diameter of each CNT fibre can also be estimated from the SEM images. The height and width of each CNT measured in Figure 8ei, Figures S1ei and S2ei also allow us to determine the approximate diameter of the CNT fibre using the following equation from Gibson et al. [34] and/or Wang et al. [35]

*D_tip_ = W^2^/4h* (1)

where *D_tip_* is the diameter of the CNT fibre, *W* is the width of the imaged CNT and can be determined from a cross section as seen in Figure 8ei, Figures S1ei and S2ei, *h* is the height of the imaged CNT and can, again, be determined from a cross section as seen in Figure 8ei, Figures S1ei and S2ei. Equation (1) assumes that the structure being imaged is much smaller than the diameter of the tip acquiring the image and is a reasonable assumption in these cases since we approximate the diameter of the surface nanotubes from their measured heights which is typically 2–7 nm compared to the measured diameter of the CNT fibres via SEM of 19–40 nm. The results of comparing the two methods for determining the CNT fibre diameter can be seen in Table 1

The results in Table 1 are interesting as there appears to be approximately a factor of 2–3 difference between the SEM analysis and Equation (1). Possible reasons for this could be due to the tapping force applied to the CNT on the surface causing it to deform slightly. This would increase *W* and decrease *h* in Equation (1) which would overall increase *D_tip_*. Another, and perhaps more significant reason, is due to the deformation of carbon nanotubes by surface van der Waals forces. Hertel et al. [36] demonstrated that the van der Waals interaction between CNTs and a surface can result in high binding energies. CNTs on a substrate may therefore experience radial and axial deformations which would result in an effective increase in measured width and decrease in measured height for the CNTs. They found that the deformation could be as high as 42% depending on the CNT diameter and number of shells. Such deformations of CNTs on a substrate will decrease the *h* and possibly increase *W* of the surface nanotube. Any decrease in measured height will result in an increase in *D_tip_* but also any increase in measured width will also be significant, especially considering *D_tip_* ∝ *W^2^*. These affects in combination could cause the discrepancy seen between the results in Table 1. Therefore the authors would recommend exercising some caution when using CNTs as a standard to measure AFM tip diameter. Hertel et al. [36] suggest that multi-walled CNTs with small diameters will experience the least deformation due to surface Van der Waals forces (e.g., (40, 40) CNTs with 8 shells). Comparison of the measured *D_tip_* between the three CNT probes and an as-received Si AFM probe without CNT attachment reveals that the CNT probes produce a significantly sharper tip. Again, the measured *D_tip_* is significantly higher than expected and further reiterates the likely overestimation of *D_tip_* using this method. However the difference between the *D_tip_* measurements can still provide information on the relative sharpness of each probe *D_tip_* was measured from multiple sections on obtained AFM images and all data is present in box plots in Figure S3.

Not all CNT fibre probes were successfully stabilised using the method outlined in Section 4.4. Tip 4 from Figure 1 was processed using high-force tapping but no images could be acquired. Closer inspection of the CNT after processing can be seen in Figure S4b and a small loop can be observed at the end of the CNT fibre. Obviously no amount of imaging or processing could dislodge or shorten this structure and this CNT fibre is therefore unusable for AFM measurements indicating that the stabilisation method shown in this work is not 100% successful but was found to be effective in ~80% of cases.

## 3. Discussion

**Figure 9:**
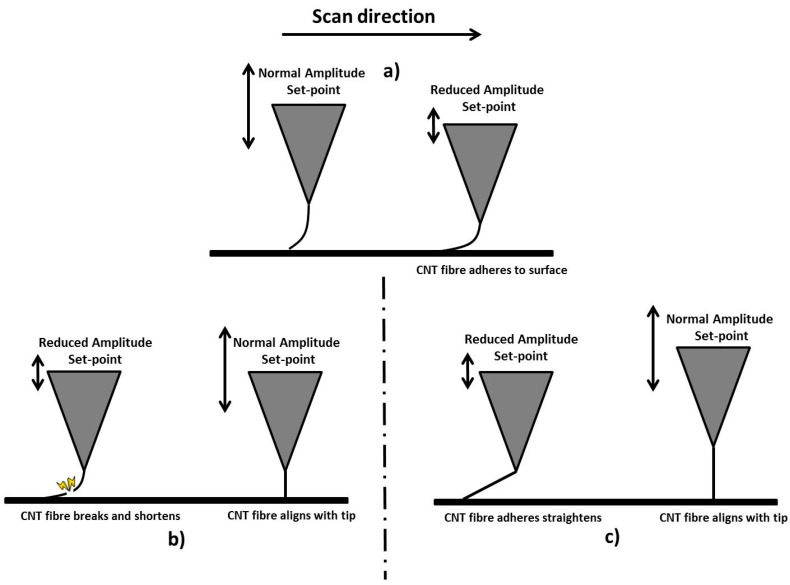
(**a**) Shows an AFM tip with a misaligned CNT fibre attached and in feedback with the surface with a normal operating tapping mode amplitude set-point. This is then reduced to 10–30% of the cantilever free amplitude which effectively presses the CNT fibre into the surface resulting in some level of adhesion. Two proposed processes are then shown in (**b**,**c**); (**b**) shows that the CNT adhesion with the surface could result in the CNT fibre breaking which results in a shorter aligned fibre once normal amplitude set-point operation is resumed. In (**c**) the adhesion results in a straightening of the CNT fibre which then aligns with the tip once normal amplitude set-point operation is resumed.

Solvent-based deposition methods have been shown to be excellent at attaching CNT fibres to AFM tips and also tungsten tips. While DEP has been used and reported before, the solvent evaporation method shown here is simpler and can be used to not only produce very long macroscopic CNT fibres (greater than 400 microns in length, see Figure 4) which may have a number of applications but can also attach much shorter CNT fibres to AFM tips (Figure 6). While many solvent-based depositions results in attached CNT fibres, many are not ready to image using AFM until some kind of stabilisation has been applied. The mechanism of the stabilisation effect is unclear, as one would expect a misaligned fibre to be bent further out of alignment by high force imaging. A possible explanation is that during the compression and relaxation of the nanotube fibre in the course of imaging; adhesion causes the tip of the fibre to stick to the surface to some degree. This would result in a “pulling” force that could stretch the fibre in the direction of the force exerted by the AFM probe, possibly causing alignment with the tip apex. The shortening effect observed for some tips is perhaps simpler than this and would either be caused by breakage of the fibre, or sliding and compression of the nanotubes within the fibre to produce a shorter, denser and hence more rigid structure. This process is described schematically in Figure 9.

As mentioned, FIB irradiation can be used to align CNT fibres as reported by Shin et al. [18] and Raghuveer et al. [19]. This provides excellent control over the orientation of the fibre, but is quite destructive. Both Shin et al. [18] and Raghuveer et al. [19] observed that TEM images of the CNT after FIB irradiation showed significant damage to the graphitic structure and substantial implantation of metal ions. While the method of CNT stabilisation described herein cannot afford the same precise control for orientation, it is far less destructive. This will be a significant advantage for applications where maintaining the graphitic structure of the CNTs is critical. Another significant advantage of the stabilisation process described in this work is that it is much simpler to apply since it only requires an AFM that can operate in tapping mode. The method can also be applied to any CNT AFM tip regardless of the attachment method and therefore will allow CNT tips that appear unusable to be resurrected.

## 4. Materials and Methods

### 4.1. SWCNT Solutions

10 mg of SWCNTs (P3, Carbon Solutions) and 0.13 g tetraoctylammonium bromide (TOAB) (Sigma-Aldrich, Castle Hill, Australia, 98%) were dissolved in 25 mL tetrahydrofuran (THF) (Sigma-Aldrich, Castle Hill, Australia, 99.9%) by sonication for 30 min. After centrifugation of the resultant solution at 10,000 rpm for 10 min, the supernatant was discarded and the precipitate was re-dispersed in THF by sonication and this process repeated to remove unbound TOAB.

### 4.2. Production of CNT Fibre Probes Using Dielectrophoresis

While the simplest application of DEP is for spherical particles, interesting effects are observed for particles with high aspect ratios. Rod-like particles such as nanowires or CNTs are found to orientate with the electric field lines, which enables alignment and deposition onto electrodes in solution. The sharp AFM tip provides a convenient electrode with which to form a non-uniform electric field, and several groups have used DEP to attach CNTs onto AFM tips in solution and studied the effect of parameters such as electric field frequency on the attachment efficiency.

**Figure 10:**
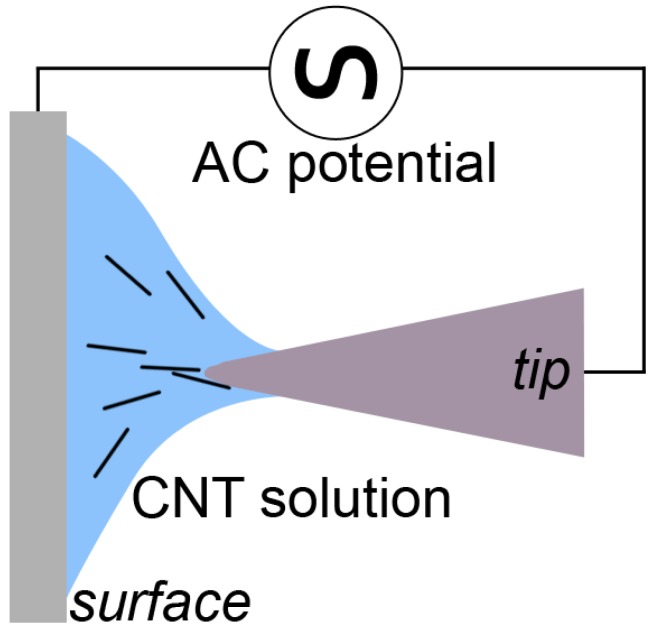
Schematic showing the assembly of CNTs from solution onto the AFM tip using dielectrophoresis.

AFM tips were aligned as shown in Figure 10, where the tip is brought to within 20 µm of a silicon wafer using an optical microscope and a unidirectional micro-translator. A droplet of the THF CNT solution as described in Section 4.1, was placed onto the silicon surface, such that a meniscus formed, bridging the gap between the tip and the surface. An alternating current (1 MHz) electrical bias (2.5 V) was then applied across the gap. The bias was applied for 20 s, and then the tip was retracted from the solution and analysed.

### 4.3. Solvent Evaporation

Evaporation of the meniscus of a CNT solution over a sharp tip was found to deposit a CNT fibre, resembling that produced by DEP. This technique was used to attach short, well-aligned fibres to atomic force microscope (AFM) probes by utilising the snap-off effect observed when the meniscus recedes over the tip. As mentioned, producing macroscopic CNT fibres could have many applications such as flexible conductors for textiles and batteries, mechanical actuators for muscles [33] and field emission sources for vacuum electronic instruments such as SEMs [21]. A similar approach was used by Ondarcuhu and Joachim to draw gold nanoparticle fibres up to 1 mm in length and with diameters of 2–100 nm [24]. The work presented here builds on this by extending the technique to SWCNT solutions.

Tungsten tips were fabricated by etching 0.25 mm tungsten wire in a 1 M sodium hydroxide (ChemSupply, Adelaide, Australia, 98%) solution, these tips were rinsed with water and were then dried in a stream of nitrogen. For both the tungsten tips and the AFM probes, the tip was brought close to the surface and then a 5 µL droplet of the SWCNT solution was applied. When drawing very long fibres a 5 µL droplet of the SWCNT solution was applied to the silicon surface and the tip was immersed in the droplet using a translator. The tip was then slowly removed from the droplet and a thin black fibre was drawn out of the solution, and attached to the apex of the tip.

### 4.4. AFM Analysis and CNT Fibre Processing

All of the AFM measurements were conducted using tapping mode in air in ambient conditions on a Multimode 8 system with Nanosocope V controller. AFM data was analysed using the Nansocope analysis software version 8.1 (Bruker Corporation, Billerica, MA, USA) with typical analysis involving the flattening of images and using the section tool to obtain cross-sections on images. AFM tips used in this work were Mikromasch silicon NSC15 probes with nominal spring constants and tip diameters of 42 N/m and 20 nm respectively. The scanner used was a Bruker J-scanner and it was calibrated in *x*, *y* and *z* directions using silicon calibration grids (Bruker model number VGRP: 10 µm pitch, 180 nm depth and Mikromasch TGZ01: 3 µm pitch, 20 nm depth).

CNT fibre straightening was performed with the cantilever tuned to 500 mV free amplitude (~20 nm) far from the surface (>200 microns). Initial imaging amplitude set-point values were typically 70 to 80% of the free amplitude (i.e., 350 to 400 mV) and then the amplitude set-point was reduced iteratively while scanning the surface until stable operation was achieved which was typically anywhere between 10 and 30% of the free amplitude (i.e., 100 to 250 mV). The time taken for the CNT in each instance to stabilise was typically 1 to 20 min. The amplitude set-point was then returned to a value typical of normal tapping mode operation (i.e., 350 to 400 mV), and imaging continued as normal. In some cases some additional images were acquired to allow the CNT fibre to “settle” into a stable configuration which could produce further improvements in image quality. Therefore the entire process could take anywhere from 10 to 45 min to complete. 

The samples imaged for fibre straightening were either the silicon calibration grid (Bruker model number VGRP: 10 µm pitch, 180 nm depth) or a silicon surface covered in CVD grown SWCNTs and MWCNTs. The procedure to produce the CVD grown CNTs was achieved by sputtering 10 nm aluminium (Proscitech, Kirwan, Australia, 99.99%) and 5 nm iron (Goodfellow, Huntingdon, UK, 99.5%) sequentially onto a bare silicon surface (Siltronix, Archamps, France, <100>, single side polished). The sample is then placed into the CVD system under a flow of 1500 sccm argon (99.997%, BOC, Adelaide, Australia) and 500 sccm hydrogen (99.98%, BOC) and then raised to 750 °C and left for 10 min once the temperature is reached. CNT growth begins by adding 200 sccm acetylene (98%, BOC) and introducing water vapour with a 2500 sccm argon flow through a water bubbler. The growth is stopped after 10 min by stopping the acetylene, hydrogen, and water vapour flow in the order listed, and the system is cooled under the original 1500 sccm flow of argon.

### 4.5. SEM Analysis

All of the SEM measurements were acquired using a CamScan MX2500 SEM. The instrument was calibrated using silicon calibration grids (Bruker model number VGRP: 10 µm pitch, 180 nm depth and Mikromasch TGZ01: 3 µm pitch, 20 nm depth). Uncertainty in instrument calibration is included in the uncertainty quoted for dimensional measurements by SEM.

## 5. Conclusions

CNT modified probes were prepared by dielectrophoresis and solvent evaporation. To the best of our knowledge, this is the first time solvent evaporation has been used for this purpose. CNT modified probes that were produced using these methods showed very high aspect ratio and decent alignment. AFM imaging with the prepared CNT-modified probes was initially impossible for many probes due to poor stability of the CNT fibres. However, we developed a method to straighten, shorten and strengthen the CNT fibre thorough AFM imaging with low amplitude set-point. Using the stabilisation procedure we prepared cost-efficient CNT modified probes capable of imaging with high resolution. 

## Figures and Tables

**Table 1:** A comparison of the measured diameter of the attached CNT fibres to tips 1–3 from Figure 1 using SEM analysis and Equation (1) which relies on AFM imaging.

AFM Tip	Diameter of Attached CNT Fibre Measured Using SEM (nm)	Diameter of Attached CNT Fibre Determined Using Equation (1) (nm)
Tip 1	19	63 ± 9.2
Tip 2	31	78 ± 27
Tip 3	40	94 ± 20
Without CNT	-	112 ± 40

## Supplementary Materials

The following are available online at www.mdpi.com/2079-4991/7/11/346/s1, Figures S1–S4.
