# Supplementary material for: Solution Based Methods for the Fabrication of Carbon Nanotube Modified Atomic Force Microscopy Probes
Source: Nanomaterials (Basel). 2017 Oct 25;7(11):346. doi: 10.3390/nano7110346 (PMC5707563; doi:10.3390/nano7110346)
Supplement: Supplementary file 1 [file nanomaterials-07-00346-s001.pdf]

Supplementary Material

Solution based methods for the fabrication of carbon nanotube modified atomic force microscopy probes

Ashley D. Slattery [1,2], Cameron J. Shearer [1,*], Joseph G. Shapter [1], Jamie S. Quinton [1] and Christopher T. Gibson [1,*]

[1] Flinders Centre for NanoScale Science and Technology, College of Science and Engineering, Flinders University, Bedford Park, SA 5042, Australia; Ashley.slattery@adelaide.edu.au (A.D.S.); joe.shapter@flinders.edu.au (J.G.S.); jamie.quinton@flinders.edu.au (J.S.Q.)

[2] Adelaide Microscopy, The University of Adelaide, Adelaide, SA 5005, Australia

* Correspondence: cameron.shearer@flinders.edu.au (C.J.S.); christopher.gibson@flinders.edu.au (C.T.G.); Tel.: +61 8 8201 2372 (C.J.S); Tel.: +61 8 8201 7978 (C.T.G); Fax: +61 8 8201 2905(C.T.G.)

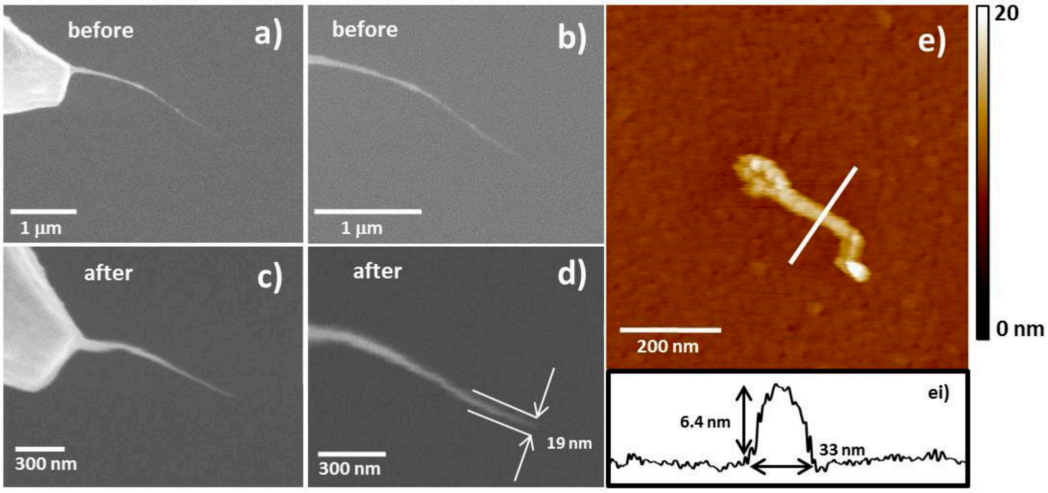

Figure S1: SEM images of tip 1 from figure 1 before processing (**a** and **b**) and after processing (**c** and **d**). Figure S1**e** is an AFM image of a CNT on a CNT covered silicon surface using tip 1 after processing with figure S1**ei** showing a cross section which corresponds to the white line in figure S1**e**. Comparing the before and after SEM images indicates there has been some change in the position of the CNT fibre which indicates some slight straightening.

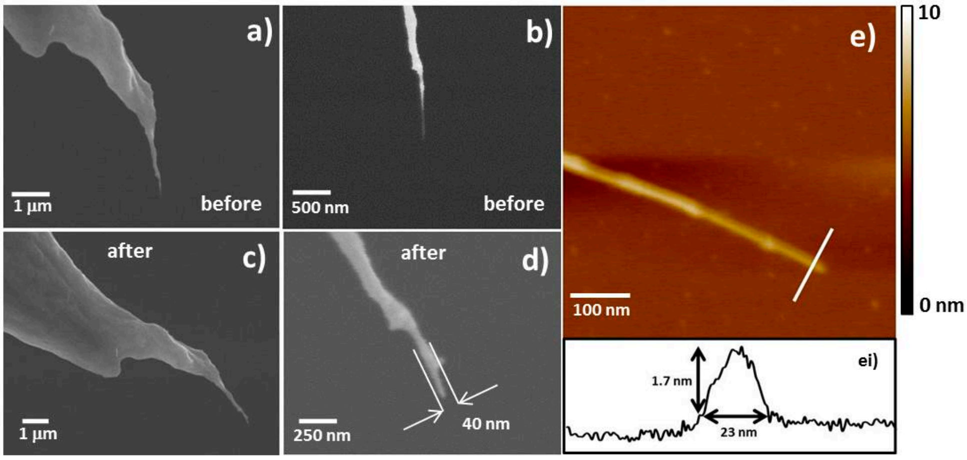

Figure S2: SEM images of tip 3 from figure 1 before processing (**a** and **b**) and after processing (**c** and **d**). Figure S2**e** is an AFM image of a CNT on a CNT covered silicon surface using tip 3 after processing with figure S2ei showing a

cross section which corresponds to the white line in figure S2e. Comparing the before and after SEM images indicates there has been a shortening of the CNT fibre.

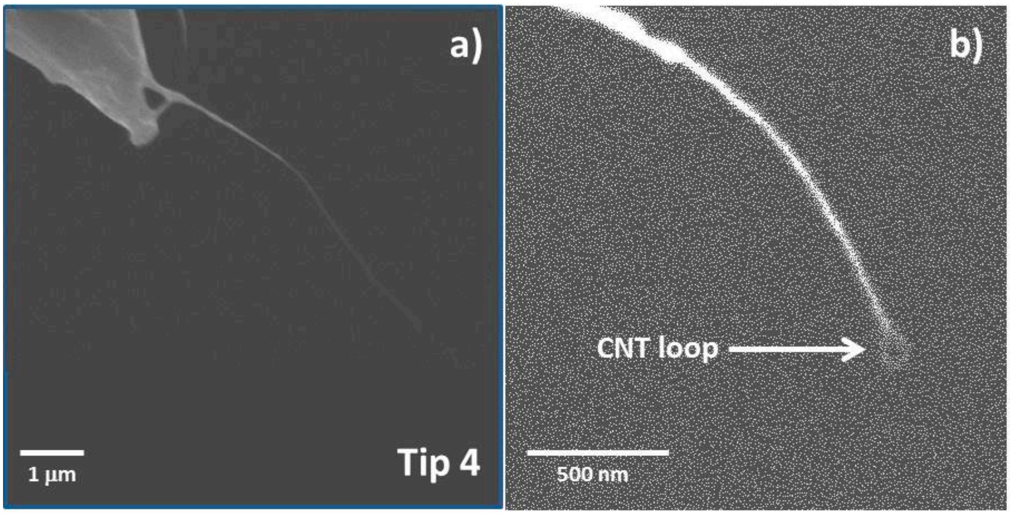

Figure S3: SEM images of tip 4 from figure 1 before processing (**a**) and after processing (**b**). No stabilisation of the CNT fibre was possible indicating that the high-force tapping method is not 100% effective. Interestingly the CNT fibre seems to be looped at the very end, as can be seen in figure S3b, which is most likely the reason that stabilisation was not possible.
